# Carotid Artery Endarterectomy Effect on Choroidal Thickness: One-Year Follow-Up

**DOI:** 10.1155/2018/8324093

**Published:** 2018-12-18

**Authors:** Gilad Rabina, Dana Barequet, Michael Mimouni, Yefim Rabinovitch, Yehuda Wolf, Adiel Barak, Anat Loewenstein, Shulamit Schwartz

**Affiliations:** ^1^Division of Ophthalmology, Sourasky Medical Center, Affiliated with Sackler School of Medicine, Tel Aviv University, Tel Aviv, Israel; ^2^Department of Ophthalmology, Rambam Health Care Campus, Affiliated with the Ruth Rappaport Faculty of Medicine, Technion-Israel Institute of Technology, Haifa, Israel; ^3^Department of Vascular Surgery, Sourasky Medical Center, Affiliated with Sackler School of Medicine, Tel Aviv University, Tel Aviv, Israel

## Abstract

**Purpose:**

To evaluate the change in choroidal thickness after carotid artery endarterectomy (CEA) in patients without retinal pathology.

**Methods:**

A prospective series of patients who underwent CEA at the Tel Aviv Medical Center. Spectral domain optical coherence tomography (SD-OCT) was performed one day before the CEA and at least 6 months after. Data included medical history, smoking history, percentage of carotid stenosis before and after CEA, best-corrected visual acuity (BCVA), central macular thickness (CMT), and choroidal thickness (subfoveal, 500 *µ*m, 1000 *µ*m, and 1500 *µ*m nasal and temporal).

**Results:**

Eight patients (seven male and one female) with a mean age of 70.5 ± 6.1 years were included in the study. The mean internal carotid artery (ICA) stenosis was 89.8% ± 5.1 in the operated side, 33.7% ± 10.9 in the nonoperated side (*p* < 0.0001), and 0% after CEA (*p* < 0.0001). Operated side BCVA was 0.35 ± 0.66 compared to 0.61 ± 0.83 in the nonoperated side (*p*=0.51). The mean subfoveal choroidal thickness (SFChT) of the operated side was 277 ± 67 *µ*m compared to 268 ± 71 *µ*m in the nonoperated side (*p*=0.81). SFChT and CMT after CEA were 275 ± 64 *µ*m (*p*=0.96) and 268 ± 29 *µ*m (*p*=0.98), respectively.

**Conclusions:**

SFChT and CMT in patients without retinal or choroidal pathology and significant ICA stenosis can be normal and may not change after successful ipsilateral CEA.

## 1. Introduction

Atherosclerotic carotid disease is an important cause of cerebrovascular accidents (CVAs) and transient ischemic attack (TIA). Furthermore, it may lead to ischemia in areas downstream to the stenotic lesion. Previous studies showed that a 90% carotid stenosis can reduce the ipsilateral central retinal artery perfusion pressure by approximately 50% [1–4] and as the degree of internal carotid artery (ICA) stenosis increases, the flow in ophthalmic artery decreases. In severe stenosis, the flow is not detectable or a reversed flow may be present [5–7]. Amaurosis fugax (AF), central retinal artery occlusion, and ocular ischemic syndrome (OIS) are among the problems of carotid disease and may eventually lead to permanent blindness [1, 8, 9]. Doppler ultrasonography is used for the measurement of blood flow in the carotid arteries; many techniques have been developed for the measurement of ocular blood flow and velocity. Though perfusion of both the ophthalmic artery and central retinal artery can be measured in experienced hands [10], this may be less feasible in standard clinical settings [9].

In the case of moderate to severe stenosis of carotid arteries and ischemic diseases of the brain such as CVA or TIA, carotid artery surgery is the most effective method of treatment [11, 12]. Disappearance of AF, decreased neovascularization of the optic nerve head and the iris, disappearance of paresis of the pupil muscle, and improvement of blood flow in orbital vessels have all been reported following carotid artery endarterectomy (CEA) [13–16]. In OIS, CEA has been reported to lead to subjective improvement in visual acuity and periorbital pain and improvement or complete resolutions of signs of ischemia in funduscopic examination [17].

Ultrasonic studies have revealed a significant decrease of choroidal thickness in patients with hemodynamically significant stenosis of the carotid arteries [18]. Other studies, using optical coherence tomography (OCT) images, have shown that affected eyes with OIS showed thinner subfoveal choroidal thickness than unaffected contralateral eyes [18–23], meaning that subfoveal choroid thinning represents impaired choroidal circulation in patients with OIS.

The purpose of this study was to assess choroidal and central macular thickness before and after CEA surgery.

## 2. Materials and Methods

This is a prospective case series of patients who underwent CEA between 4/2015 and 12/2015 in the Tel Aviv Medical Center. According to conventional guidelines, CEA was performed in the presence of ICA stenosis of 50% to 99% [12, 24].

The study adhered to the tenets of the Declaration of Helsinki and was approved by the Institutional Review Board (IRB) of the Tel Aviv Medical Center, Tel Aviv, Israel. Approval and informed consent was obtained from all patients.

### 2.1. Retinal and Choroidal Measurements

Spectral domain optical coherence tomography (SD-OCT) (Spectralis OCT; Heidelberg Engineering, Heidelberg, Germany) was performed one day before the CEA and at least 6 months after the procedure. Measurements included central macular thicknesses (CMTs) and choroidal thickness. Choroidal thickness measurement was made with the use of enhanced-depth imaging (EDI) SD-OCT. All measurements were performed between 9 and 11:30 AM in order to deal with the potential effect of diurnal variation.

**Figure 1:**
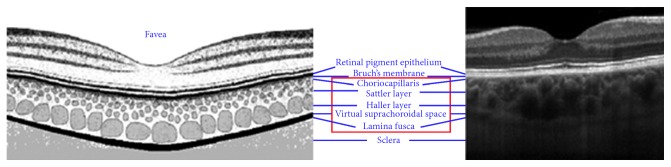
Choroid anatomy (histology and OCT). Choroid boundaries measured as the distance between the RPE/choroid interface and choroid/sclera interface. OCT: optical coherence tomography; RPE: retinal pigment epithelium.

Choroidal thickness was measured as the perpendicular distance between the hyperreflective outer border of the retinal pigment epithelial layer and the sclerochoroidal interface (Figure 1). We measured subfoveal and 500 *µ*m, 1000 *µ*m, and 1500 *µ*m nasal and temporal to the subfoveal measurement.

### 2.2. Data Collection

All patients underwent a complete ophthalmic examination, and the data collected included patients' demographics, medical history, smoking history (evaluated in pack-years, i.e., one pack-year = 20 cigarettes/day for one year), percentage of carotid stenosis before and after CEA, best-corrected visual acuity (BCVA), slit-lamp examination and fundoscopy findings, CMT, and choroidal thickness. Patients under the age of 18, with insufficient or poor-quality images, other retinal or choroidal disease (such as retinal artery occlusion, retinal vein occlusion, uveitis, age-related macular degeneration (AMD), central serous chorioretinopathy (CSR), and polypoidal choroidal vasculopathy (PCV)), macular pathologies (including epiretinal membrane, cystoid macular edema, subretinal fluid, macular hole, or atrophy), or high myopia, were excluded. The main outcome was change in choroidal thickness before and after CEA.

### 2.3. Data Analysis

BCVA was recorded using a Snellen chart and was converted to the logarithm of minimal angle of resolution (logMAR) value for statistical purposes. When dealing with variables that were not normally distributed, the Mann–Whitney test was used. Student's *t*-test was used to compare parameters between groups. Data were recorded in Microsoft Excel (2010) ™ and analyzed using SPSS version 23 (SPSS Inc., Chicago, IL, USA). All tests were 2-tailed, and the threshold for statistical significance was defined as a *p* value < 0.05. Continuous variables are described as mean ± standard deviation.

## 3. Results

A total of 12 patients were recruited, two passed away and two were lost during the follow-up period. A total of eight patients (seven male and one female) completed the follow-up and were included in this study.

### 3.1. Patient Characteristics

The mean age of the patients was 70.5 ± 6.1 (range 61–77) years. All the patients suffered from hypertension (HTN) and had a medical history of CVA or TIA. Most of the patients were smokers and had a history of hypercholesterolemia. Five patients had right-side and three left-side significant ICA (*p*=0.35). Mean ICA stenosis was 89.8% ± 5.1 in the operated side, compared to 33.7% ± 10.9 in the nonoperated side (*p* < 0.0001) and to 0% after CEA (*p* < 0.0001). Operated side BCVA was 0.35 ± 0.66 compared to 0.61 ± 0.83 in the nonoperated side (*p*=0.51). The lower vision in the nonoperated side was attributed to hand motion and 6/30 and 6/60 vision in three patients due to cataract and absent posterior segment morbidities. The characteristics and demographic features of the patients are demonstrated in Tables 1 and 2.

### 3.2. Choroidal and Retinal Measurements

The mean subfoveal choroidal thickness (SFChT) of the operated side was 277 ± 67 *µ*m compared to 268 ± 71 *µ*m in the nonoperated side (*p*=0.81). When comparing the choroidal thickness measurements of the operated side to the nonoperated side in six other locations such as 500 *µ*m, 1000 *µ*m, 1500 *µ*m nasal and 500 *µ*m, 1000 *µ*m, and 1500 *µ*m temporal relative to the SFChT measurement, we did not find any statistically significant difference (*p* = 0.80, 0.92, 0.52, 0.63, 0.89, 0.75, respectively). CMT of the operated side was 267 ± 31 *µ*m compared to 271 ± 19 *µ*m in the nonoperated side (*p*=0.76) (Table 3).

The second measurements of the SFChT were performed at 14.3 ± 4 (range 6–19) months after the first one. At the time of the second measurements, average ICA stenosis was 0% in the operated side (*p* < 0.0001). SFChT after CEA was 275 ± 64 *µ*m (*p*=0.96). When comparing the choroidal thickness measurements, before and after CEA, in the other six locations as previously described, no statistically significant difference was found (*p* = 0.85, 0.97, 0.74, 0.95, 0.91, 0.78, respectively). CMT after CEA was 268 ± 29 *µ*m (*p*=0.98) (Table 4).

**Figure 2:**
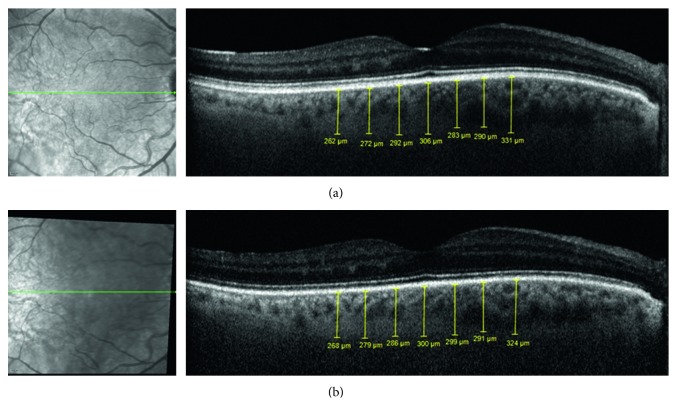
Patient #1: a 61-year-old male, with a past medical history of hypertension, hypercholesterolemia, TIA, and smoking (40 pack-years), presented with right ICA stenosis of 80%. His BCVA at presentation was RE 6/6.5 and LE 6/7.5. Mild nuclear sclerosis was present in both eyes with no pathological findings when performing fundoscopy of both eyes. He underwent an uneventful CEA, and on follow-up, there was no right ICA stenosis at all. EDI SD-OCT images of the RE, with choroidal thickness measurements (subfoveal; 500 *µ*m, 1000 *µ*m, and 1500 *µ*m nasal and temporal), obtained one day before right CEA (a) and 6 months after CEA (b). TIA: transient ischemic attack; ICA: internal carotid artery; BCVA: best-corrected visual acuity; RE: right eye; LE: left eye; CEA: carotid artery endarterectomy; EDI: enhanced depth imaging; SD-OCT: spectral domain optical coherence tomography.

**Figure 3:**
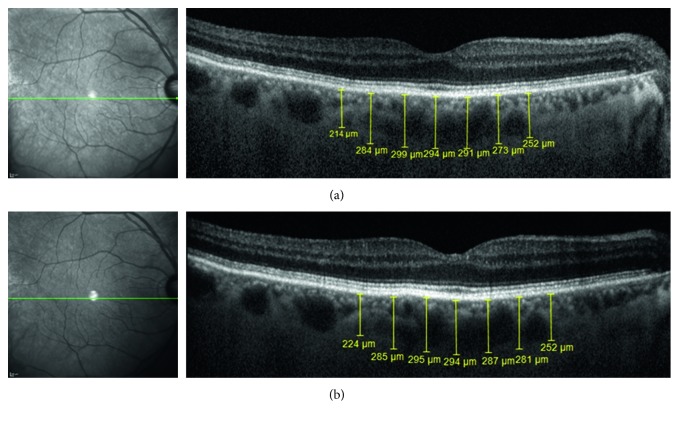
Patient #7: a 75-year-old male, with a past medical history of hypertension, CVA, CAD, hypercholesterolemia, and smoking (40 pack-years), presented with right ICA stenosis of 90%. His BCVA at presentation was RE 6/7.5 and LE 6/7.5. Moderate nuclear sclerosis was present in both eyes with no pathological findings in the funduscopy of both eyes. He underwent an uneventful CEA, and on follow-up, there was no right ICA stenosis at all. EDI SD-OCT images of the RE, with choroidal thickness measurements (subfoveal; 500 *µ*m, 1000 *µ*m, and 1500 *µ*m nasal and temporal), obtained one day before right CEA (a) and 14 months after CEA (b). CVA: cerebrovascular accident; CAD: coronary artery disease; ICA: internal carotid artery; BCVA: best-corrected visual acuity; RE: right eye; LE: left eye; CEA: carotid artery endarterectomy; EDI: enhanced depth imaging; SD-OCT: spectral domain optical coherence tomography.

Figures 2 and 3 show the follow-up SD-OCT images of 2 patients.

### 3.3. Post Hoc Power Analysis

Assuming that the difference in SFChT before and after surgery would be real, we calculated at a power of 0.8 that 8,421 subjects would be required for this difference to reach the 0.05 significance level.

## 4. Discussion

ICA stenosis can cause a decrease in ophthalmic artery flow and as a result can cause ocular complications, including AF, central retinal artery occlusion, and OIS [3, 4, 25]. There is sufficient evidence to suggest that CEA has a positive effect for patients with OIS and that the earlier treatment is instituted the better the clinical outcome [3]. However, when to intervene and whether or not it is of benefit for patients without ocular pathology is still unclear [26]. In our study, we examined patients with significant ICA stenosis but without any ocular pathology, besides cataract. All the patients in our study had severe ICA stenosis, suffered from HTN, and had a medical history of CVA or TIA. These findings correlate with previous studies which demonstrated patients with ICA stenosis suffer from other arteriovascular disease such as coronary artery disease, TIA, CVA and have a higher rate of HTN, hypercholesterolemia, and diabetes mellitus [27–31].

Previous studies show that SFChT in the normal population varies between 260 and 300 *µ*m [32–36]. There are several conditions and diseases that can cause a change in choroidal thickness such as AMD and myopia that result in thinner choroid or PCV and CSR that result in thicker choroid [37–45]. In our study, at presentation, we found SFChT of 277 ± 67 *µ*m in the operated side compared to 268 ± 71 *µ*m in the nonoperated side (within the range of normal SFChT). This finding was surprising, as not only did we not find a thinner SFChT on the operated side but it was slightly thicker. Only few studies examined SFChT in the presence of ICA stenosis, and they showed contradicting findings. Akçay et al. showed similar findings to ours. They examined 21 patients with more than 70% ICA stenosis on one side and less than 70% stenosis in the fellow ICA. They found SFChT of 231 *µ*m on the stenotic side, compared to 216 *µ*m on the other side. Their theory is that the increased SFChT that was observed at the stenotic side might be a result of choriocapillaris vasculature dilatation, aimed to prevent retinal and choroidal ischemia due to the diminished blood flow caused by the ICA stenosis [21]. Contrarily, other studies found different results. Sayin et al. compared 25 patients with ICA stenosis and 25 age- and gender-matched healthy individuals and found a significantly thinner SFChT in the study group. Nevertheless, no significant correlation between the degree of ICA stenosis and the SFChT was found [22]. Wang et al. retrospectively examined 219 patients and found that mean SFChT of the ICA stenosis group was significantly lower than that of normal eyes. They concluded that ICA stenosis can influence the blood flow of the posterior ciliary arteries, which results in an insufficient blood perfusion of choriocapillaris [46]. Ivashina et al. found mixed results when examining 12 patients with significant ICA stenosis and found the choroid thickness was reduced only in 3 patients [18]. In cases of ICA and OIS, previous studies found choroidal thickness and volume of OIS eyes were smaller than those of unaffected fellow eyes [19, 23]. OIS results from chronic ocular hypoperfusion due to stenosis [3]; thus, the results of thinner SFChT are expected.

During our follow-up, after a median period of 14 months, we did not find any change in choroidal thickness and CMT of the operated side. In this study, we focused on change in choroidal thickness and CMT after CEA for patients with normal fundoscopy findings. These findings stand in somewhat contrast to previous studies who examined the effects of carotid artery surgery on ocular blood flow. Riiheläinen et al. examined 17 patients and concluded that CEA resulted in significantly increased flow in the central retinal artery and ophthalmic artery [47]. Kobayashi et al. examined 45 patients and demonstrated an increase in retinal artery pressure [2]. Costa et al. reported 17 patients with severe ICA stenosis and found that hemodynamic changes in patients undergoing CEA suggest improvement in the ipsilateral retrobulbar blood flow [25]. Other studies found an improvement in ophthalmic artery blood flow and improvement in visual acuity for patients with OIS after CEA [17, 48–50].

A potential theory is that, in patients with ICA stenosis but without OIS or other ocular ischemic findings, the ocular blood flow can be reduced or normal, depending on the ophthalmic artery blood flow. The ophthalmic artery blood flow (and not the upstream carotid artery blood flow) before CEA will determine the improvement in ocular perfusion after CEA. If there is a decrease in ophthalmic artery blood flow due to ICA stenosis, it will improve after CEA [5–7] and will result in an increase in choroidal thickness. If there is no decrease in ophthalmic artery blood flow, there will be no change in choroidal thickness after CEA. We speculate that, in our patients who had normal choroidal thickness and did not demonstrate any pathological fundoscopy findings, despite significant ICA stenosis, the ophthalmic artery blood flow was sufficiently preserved. Thus, there was no change in choroidal thickness during the follow-up despite improvement in carotid artery flow. Unfortunately, this remains a theory as we did not measure the ophthalmic artery blood flow in our patients before and after CEA.

Recently, Lareyre et al. recently reported that, in patients with severe ICA without ocular symptoms or findings, choroidal thickness increased bilaterally after CEA in patients, more so on the ipsilateral side [51]. These findings contradict those of the current study where in a similar cohort of patients, no such improvement in choroidal thickness was detected. A potential explanation is that though CEA improves ocular blood flow and pressure, it does not necessarily change morphology of the blood vessels, particularly when there is atherosclerosis. Indeed Akҫay et al. previously demonstrated that there may even be a compensatory choroidal thickness increase in ipsilateral internal carotid artery stenosis greater than 70% [21]. As such, further larger prospective studies combining multiple imaging modalities may shed light on this controversial matter.

The main limitations of our study are small sample size and lack of ultrasonography of the ophthalmic artery and other ocular blood vessels. The small sample size could lead to type II error; however, assuming that the clinically insignificant difference in SFChT before and after CEA surgery (2 *μ*m) would be real, 8,421 subjects would be required for this difference to reach statistical significant, a feat that would be nearly impossible to carry out. With that being said, a larger scale cohort is needed in order to further strengthen our preliminary findings. An additional limitation of this study is that fluorescein angiography was not routinely performed before and after CEA. Similarly, axial length was not routinely measured in these patients, and as such, we cannot comment on any correlation between flow and axial length. Further studies may consider incorporating these modalities as well. Last, it may be that an increase in choroidal thickness would be easier to identify in patients with thinner preoperative choroidal thickness, and future studies may consider studying the effect of CEA on these patients.

In summary, this study reports that SFChT and CMT in patients with normal fundoscopy exam and significant ICA stenosis can be normal and may not change after ipsilateral CEA.

## Figures and Tables

**Table 1:** The demographic features and baseline characteristics of patients.

			*p* value
Number of patients	8		
Age (mean ± SD)	70.5 ± 6.1 (61–77)		
Sex, male/female	7/1		0.008
Hypertension, *n* (%)	8 (100)		
Hypercholesterolemia, *n* (%)	6 (75)		
Coronary artery disease, *n* (%)	4 (50)		
Tobacco use, *n* (%)	5 (62)		
TIA, *n* (%)	5 (62)		
CVA, *n* (%)	5 (62)		
Diabetes mellitus	3 (37)		
Endarterectomy side, right/left	5/3		0.35

	Operated side	Nonoperated side	

Carotid stenosis (%)	*89.8 ± 5.1*	*33.7 ± 10.9*	>0.0001
BCVA (logMAR)	0.35 ± 0.66	0.61 ± 0.83	0.51
IOP	15.8 ± 2.1	15.3 ± 1.8	0.62

TIA: transient ischemic attack; CVA: cerebrovascular accident; BCVA: best-corrected visual acuity; IOP: intraocular pressure.

**Table 2:** Patient characteristics.

Serial no.	Sex	Age	Side of endarterectomy	Carotid stenosis before endarterectomy (%)	Carotid stenosis after endarterectomy (%)	Carotid stenosis, nonoperated side (%)	HTN	TIA	CVA	Smoker (pack-years)	CAD	Hypothyroidism	DM	Hypercholesterolemia	COPD
1	Male	61	Right	80	0	50	+	+		+ (40)				+	
2	Male	63	Left	99	0	30	+		+	+ (50)				+	
3	Male	77	Right	90	0	25	+	+	+		+		+		
4	Male	75	Right	90	0	35	+	+	+	+ (30)	+	+		+	
5	Female	71	Left	90	0	30	+		+			+			
6	Male	76	Right	90	0	20	+	+		+ (40)			+	+	+
7	Male	75	Right	90	0	30	+		+	+ (40)	+			+	+
8	Male	68	Left	90	0	50	+	+			+		+	+	

HTN: hypertension; TIA: transient ischemic attack; CVA: cerebrovascular accident; CAD: coronary artery disease; DM: diabetes mellitus; COPD: chronic obstructive pulmonary disease.

**Table 3:** CMT and SFChT comparison between the operated and nonoperated sides.

	Operated side	Nonoperated side	*p* value
CMT (*µ*m)	*267 ± 31*	*271 ± 19*	0.76
SFChT (*µ*m)	277 ± 67	268 ± 71	0.81
Choroidal thickness (*µ*m) (relative to fovea)			
500 *µ*m nasal	267 ± 59	259 ± 66	0.8
1000 *µ*m nasal	245 ± 60	248 ± 56	0.92
1500 *µ*m nasal	292 ± 57	272 ± 63	0.52
500 *µ*m temporal	272 ± 58	258 ± 67	0.63
1000 *µ*m temporal	257 ± 71	253 ± 60	0.89
1500 *µ*m temporal	253 ± 68	243 ± 61	0.75

CMT: central macular thickness; SFChT: subfoveal choroidal thickness.

**Table 4:** CMT and SFChT before and after carotid artery endarterectomy.

	Before endarterectomy	After endarterectomy	*p* value
CMT (*µ*m)	*267 ± 31*	*268 ± 29*	0.98
SFChT (*µ*m)	277 ± 67	275 ± 64	0.96
Choroidal thickness (*µ*m) (relative to fovea)			
500 *µ*m nasal	267 ± 59	261 ± 72	0.85
1000 *µ*m nasal	245 ± 60	244 ± 76	0.97
1500 *µ*m nasal	292 ± 57	278 ± 76	0.74
500 *µ*m temporal	272 ± 58	271 ± 53	0.95
1000 *µ*m temporal	257 ± 71	261 ± 58	0.91
1500 *µ*m temporal	253 ± 68	244 ± 59	0.78

CMT: central macular thickness; SFChT: subfoveal choroidal thickness.

## Data Availability

The data used to support the findings of this study are available from the corresponding author upon request.
